# Morphological differentiation across the invasive range in *Senecio madagascariensis* populations

**DOI:** 10.1038/s41598-020-76922-5

**Published:** 2020-11-18

**Authors:** Bruno Dematteis, María S. Ferrucci, Juan P. Coulleri

**Affiliations:** Northeast Botanical Institute, Sgt. Cabral 2131, C.C. 209, 3400 Corrientes, Argentina

**Keywords:** Invasive species, Population dynamics

## Abstract

Invasive species are characterized by their ability to colonize new habitats and establish populations away from their native range. In this sense, these plants are expected to have plastic responses to adapt to the environmental pressures during the invasion process. Hence, the role of natural selection is essential because it might favor the occurrence of advantageous traits. However, gene flow can counteract natural selection because immigrants introduce genes adapted to different conditions, with these introductions tending to homogenize allelic frequencies. In this work, we explore the effect of natural selection in invasive populations of *S. madagascariensis* in Argentina. We quantified leaf area, head number, and length of internodes and inflorescence from material spanning 54 years (1962–2016) and then compared between the edge versus established ranges. Our results show differences in all the measured plant traits among the sampled areas. However, only leaf area was statistically significant, which evidences different responses under the same environmental pressures in the areas located in the edge and established ranges. On the other hand, unlike homogeneous areas, the areas characterized by phenotypically diverse individuals were related to higher dispersal ability. In this sense, long-distance dispersal between neighboring areas may have had an important role in the recorded values. Furthermore, the implications of natural selection and founder effect in the invasion of *S. madagascariensis* are discussed.

## Introduction

Biological invasions cause global changes due to their impacts on ecosystems and biodiversity^1^. In addition, international trade, transport and tourism have contributed to an exponential increase of the migration of non-native plant species worldwide^2^. During range expansion, invaders colonize new habitats and establish populations away from their native range; therefore, factors that affect spread and establishment become very important^3,4^.

To ensure its survival and colonization of the new habitat, invasive plants are expected to have plastic responses to adapt to the environment pressures faced during the invasion process^5,6^ The phenotype is the result of the interaction between genetic components, evolutionary forces and natural selection^7,8^. Hence, the role of natural selection is essential because it might favor the occurrence of advantageous traits in survival and dispersal terms^9^.

Natural selection is recognized by Darwinism as the main mechanism driving evolutionary changes, and can take three forms, depending on the favored trait: (1) directional selection, which favors one of the extreme phenotypes; (2) stabilizing selection, which favors phenotypes with mean values; and (3) disruptive selection, which favors both phenotype extremes^10^. However, Darwin also gives importance to what is currently known as genetic drift and gene flow as forces that can counteract natural selection^11^. This phenomenon tends to adapt a population to local conditions (deme), but immigrants from other populations will introduce genes adapted to different conditions, which tends to homogenize allelic frequencies. In the absence of gene flow, divergent selection could favor traits that provide advantageous results in patterns of local adaptation^9,12^. The evolution of locally adapted genotypes requires consistent geographical variation in selective regimes that cause directional trait changes, as well as limited gene flow^13^. In contrast, genetic exchange and genetic diversity could contribute to the presence of individuals with genotypes associated with a higher Darwinian fitness^14^.

*Senecio madagascariensis* Poir. (Asteraceae) is a species native to South Africa and Madagascar that is currently considered invasive in Australia^15^, south-east Asia^16^, South America^17,18^ and North America^19^. In South America, this species was recorded for the first time in Argentina, where it was found in the port area in Bahía Blanca (Buenos Aires province) in the 1940s^17^. In about 30 years, *S. madagascarienis* successfully invaded the north of Buenos Aires province and nearby provinces. Currently, this species is widely distributed in northern and central Argentina, and southeastern Brazil^20^.

**Figure 1:**
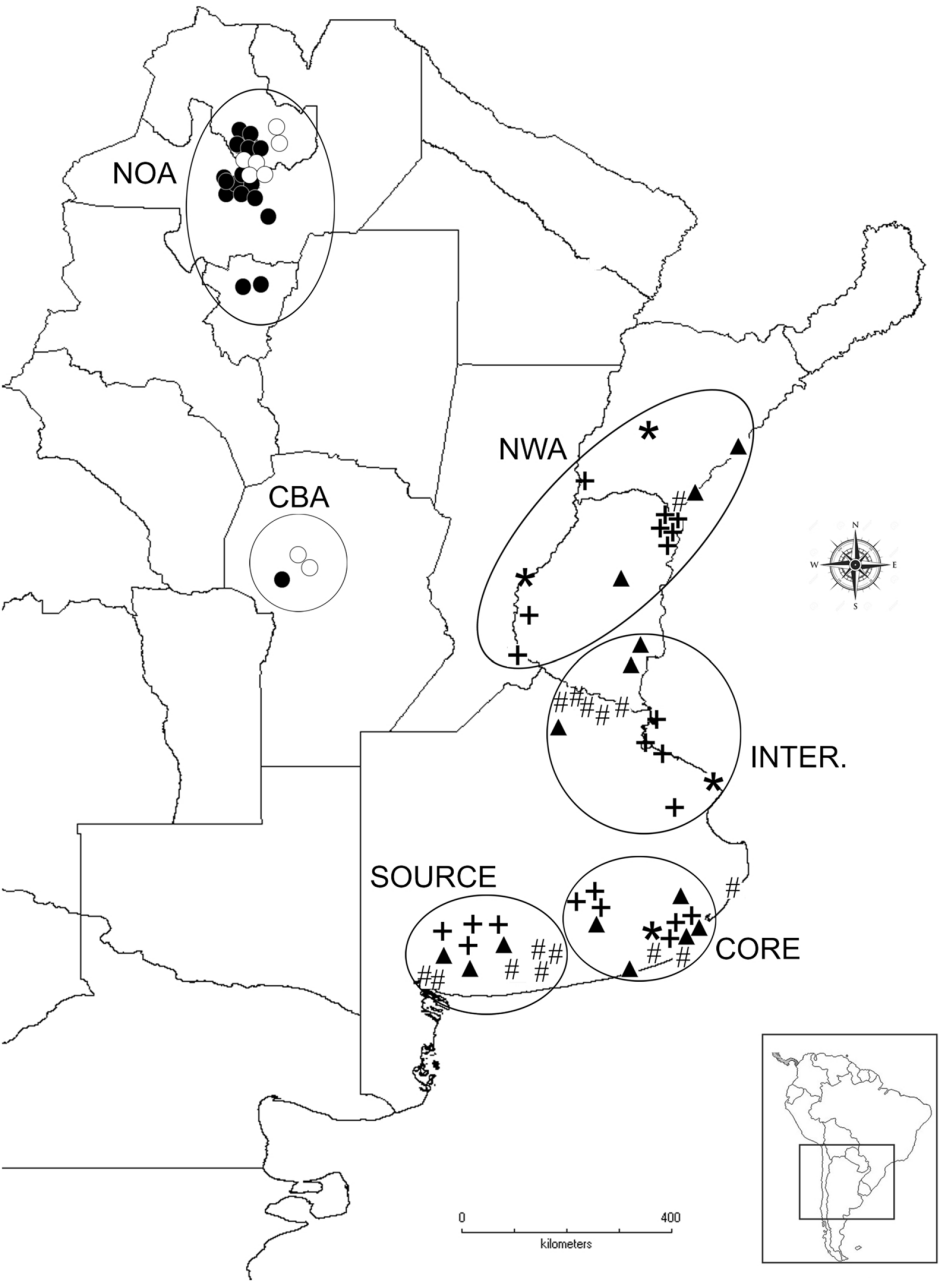
Distribution of the analyzed *Senecio madagascariensis* populations. The map was performed with Diva Gis (https://www.diva-gis.org/) software using "Administrative Layers" for Argentina. The GIS shape files were obtained from GADM database (https://www.gadm.org, version 3.6) in DIVA GIS (https://www.diva-gis.org/gData).

The studies about the rapid evolutionary changes in non-native species are of great interest because they may prove if post-introduction success is due to a genetically based change^21–23^. Indeed, identifying the phenotypic traits that might evolve to favor dispersal is an interesting topic^24^. The aim of our study was to explore the evolutionary forces that have acted to shape the invasive populations of *Senecio madagascariensis* in Argentina. We quantified life history traits in individuals obtained during field collections as well as in herbarium specimens, spanning 54 years (from the first record to the present). In addition, we analyzed how natural selection may have acted during the invasions process. Since *S. madagascariensis* has a wide distribution range, we hypothesize that natural selection may have favored individuals that invested more energy in rapid growth, increasing size and reproduction (Fig. 1).

## Results

### Plant traits

**Figure 2:**
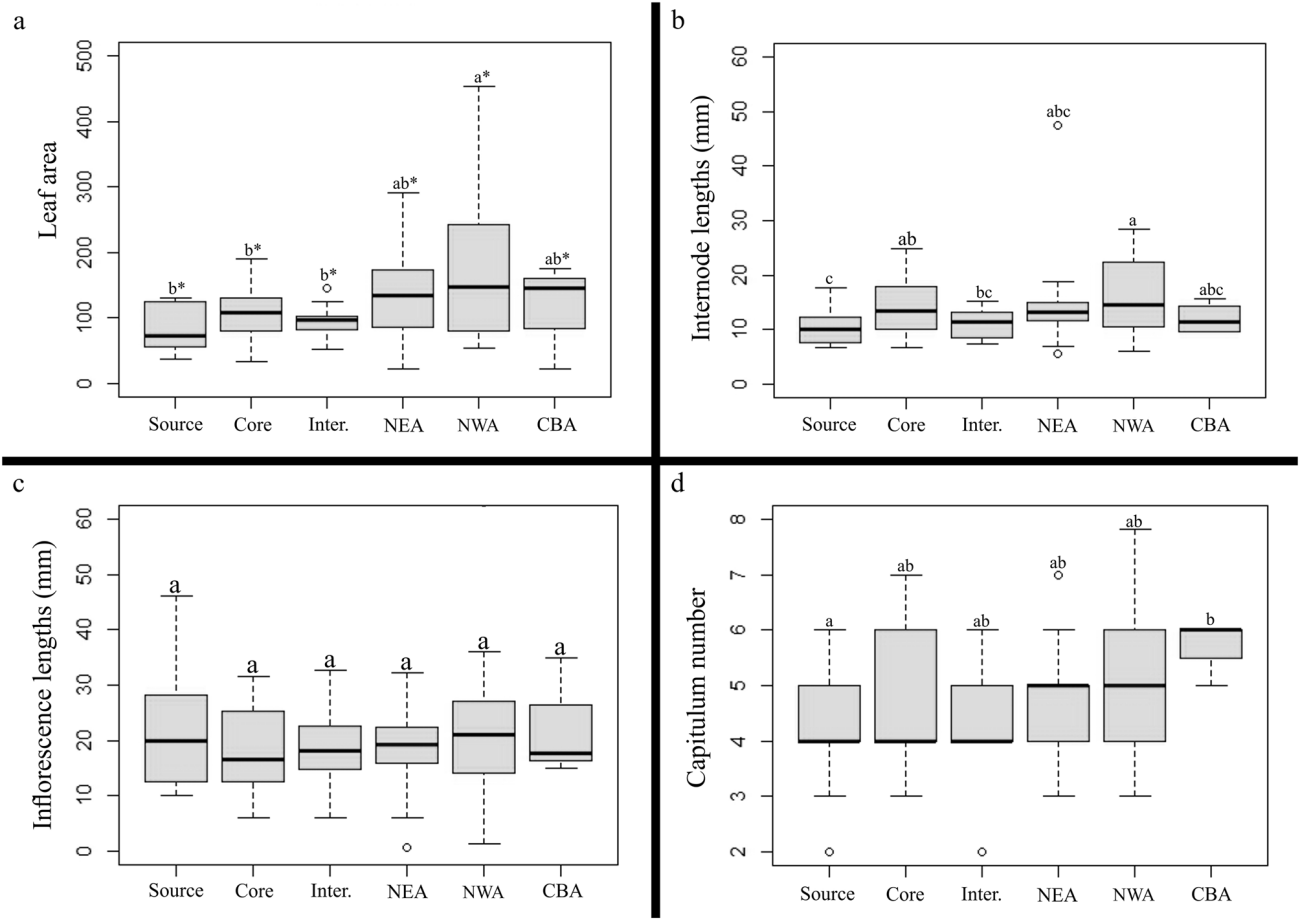
Boxplots of the traits analyzed in the sampled areas. Boxes represent s.d. and the median is shown as a line across the box. Tukey’s test (*p* < 0.05), the letters and (*) represents significant differences.

Mean values of the analyzed morphological traits are presented in Table 1 and Table S1. The NWA populations had the highest values of leaf area, and length of internode and inflorescences, whereas the lowest values were reported in the source populations (Fig. 2a–c). Only leaf area presents statistically significant differences among areas (Table 2). Regarding number of heads, the CBA populations had the highest values, whereas the source populations had the lowest ones (Fig. 2d), but without statistically significant differences (Table 2).

**Table 1:** Average values and standard deviation (SD) of the plant traits in the sampled areas.

Area	Leaf area				Internode length				Inflorescence length				Number of heads			
	Min	Mean	Max	SD	Min	Mean	Max	SD	Min	Mean	Max	SD	Min	Mean	Max	SD
Source	36.3	85.6	130.0	36.1	6.6	10.4	17.6	3.4	10.0	21.7	46.0	10.8	2.0	4.0	6.0	1.1
Core	32.8	106.7	189.5	37.5	6.6	21.6	28.9	37.9	6.0	17.7	31.5	6.9	3.0	4.7	7.0	1.2
Intermediate	51.4	94.2	145.6	27.7	7.3	11.0	15.3	2.7	6.0	19.2	32.7	7.7	2.0	4.5	6.0	1.1
NEA	22.1	167.2	294.5	164.0	5.6	14.8	47.5	9.3	0.6	18.8	32.2	8.8	3.0	4.9	7.0	1.1
NWA	53.2	189.8	462.8	174.5	6.1	15.8	28.5	7.1	1.4	22.9	36.0	15.6	3.0	5.2	7.0	1.5
CBA	22.0	121.8	174.7	68.0	9.6	12.0	15.6	2.9	15.0	21.3	35.0	9.2	5.0	5.8	6.0	0.5

**Table 2:** Results of the Tukey’s test and post-hoc multiple comparison.

Area	Foliar area			Internode length			Inflorescense length			Number of heads		
	Chisq	ms	*P*-value	Chisq	ms	*P*-value	Chisq	ms	*P-*value	Chisq	ms	*P-*value
Populations	42.69	41	0.39	59.61	41	0.03^a^	49.48	41	0.17	42.69	41	0.39
Areas	14.880	5.000	0.01^a^	9.330	5.000	0.090	1.170	5.000	0.940	7.770	5.000	0.160

Ms, mean square; Chisq, chi square value.

^a^Indicates statistically significant differences.

### Comparisons among sampled areas and populations

**Figure 3:**
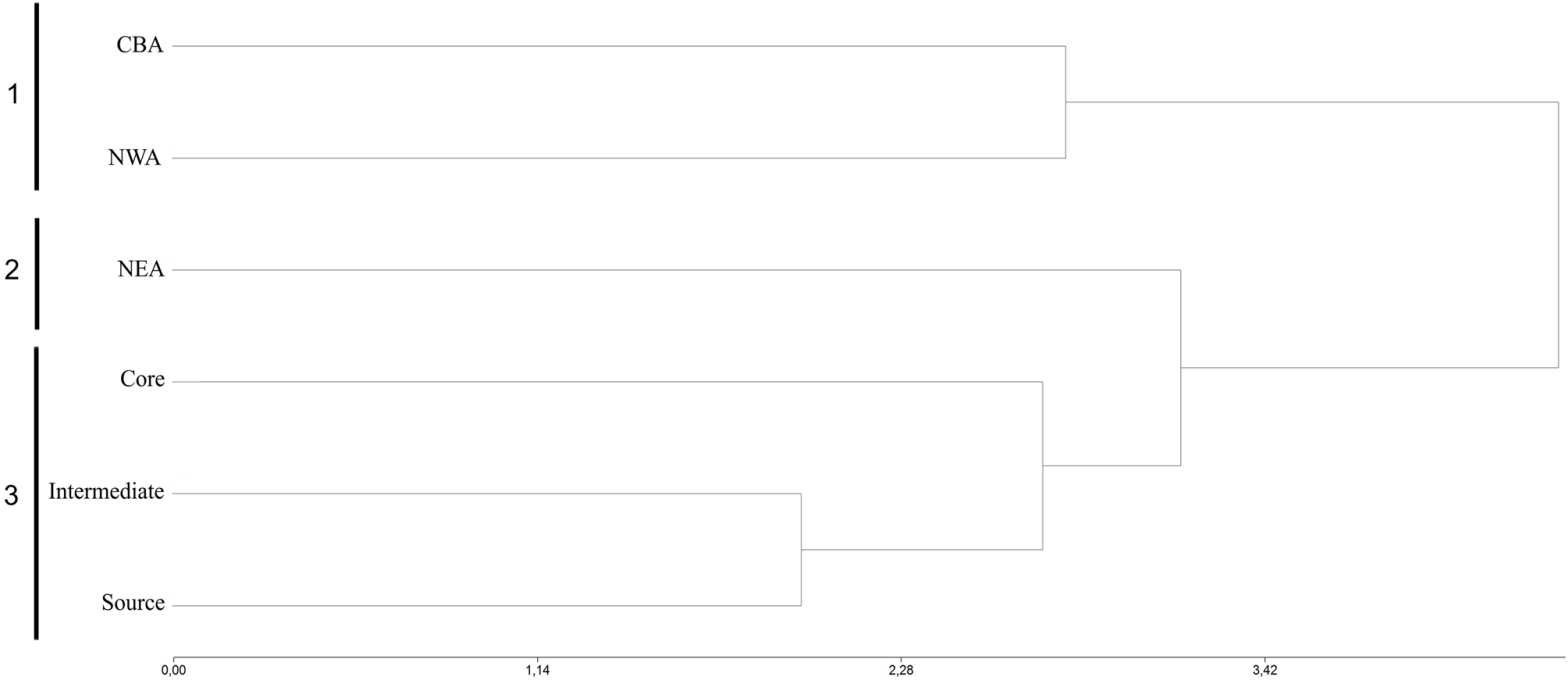
Dendrogram showing phenotypic distances among areas.

The dendrogram evidenced three area clusters (1–3), which were determined by phenotypic distances (Fig. 3). Cluster 1 included NWA and CBA, which shared number of heads. Cluster 2 involved only NEA, which had intermediate values between clusters 1 and 3 for all traits. Cluster 3 included the core, intermediate and source areas, characterized by the lowest values for most of the analyzed traits.

**Figure 4:**
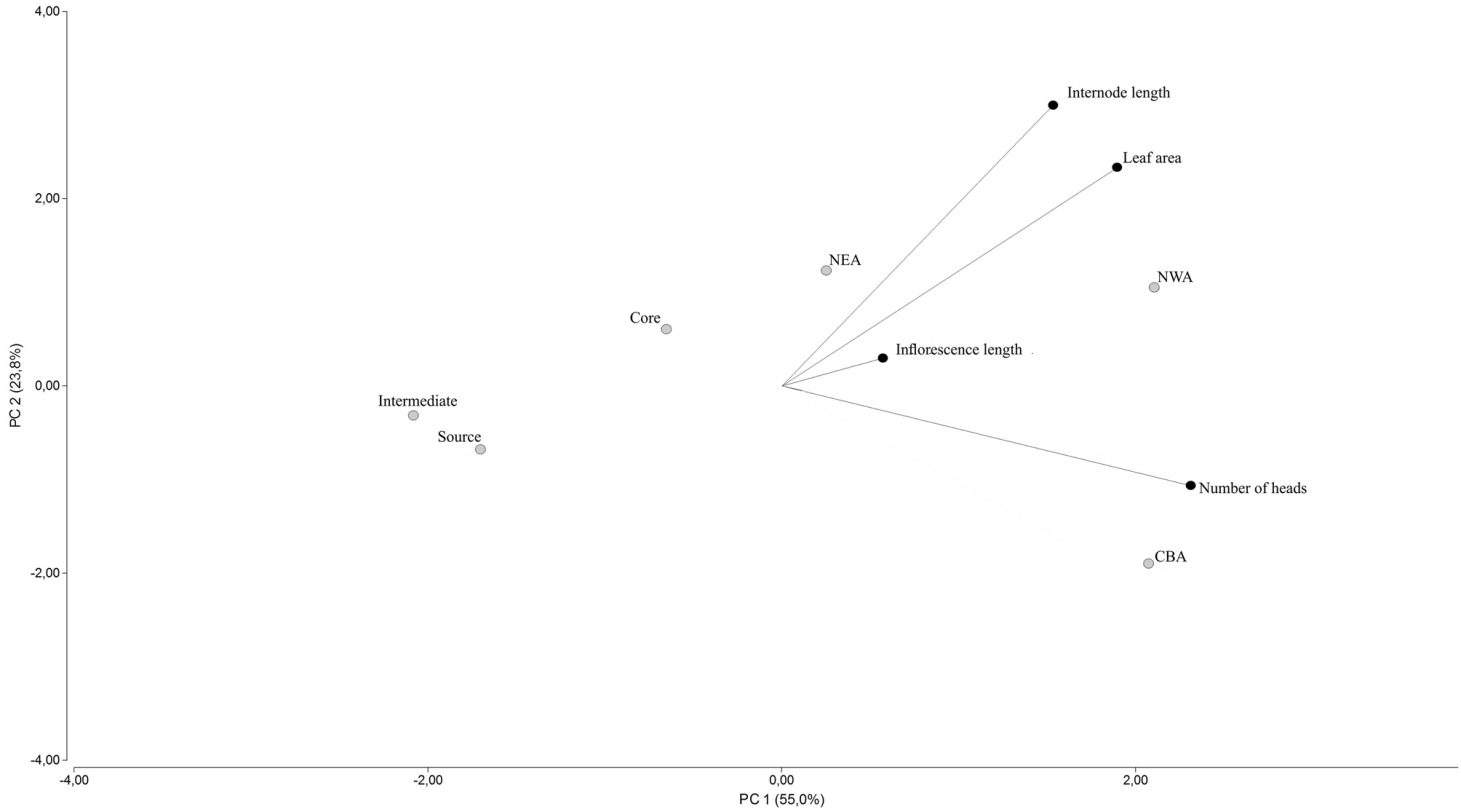
Biplot showing relationships between life history (black circles) and sampled areas (gray circles).

The PCA (Fig. 4) showed a cophenetic correlation (0.92) and the contribution of all the analyzed morphological traits, except for inflorescence length, to the differentiation between areas (Table 3). Variability of phenotypic traits (78.8%) was explained by the first two principal components (PC1, 55% and PC2, 23.8%).

**Table 3:** Contribution of plant traits to the first two components of the principal component analysis.

Variable	PC 1	PC 2
Leaf area	0.41	0.51
Internode length	0.330	0.65
Inflorescence length	0.12	0.06
Number of heads	0.51	− 0.24

### Natural selection action

**Figure 5:**
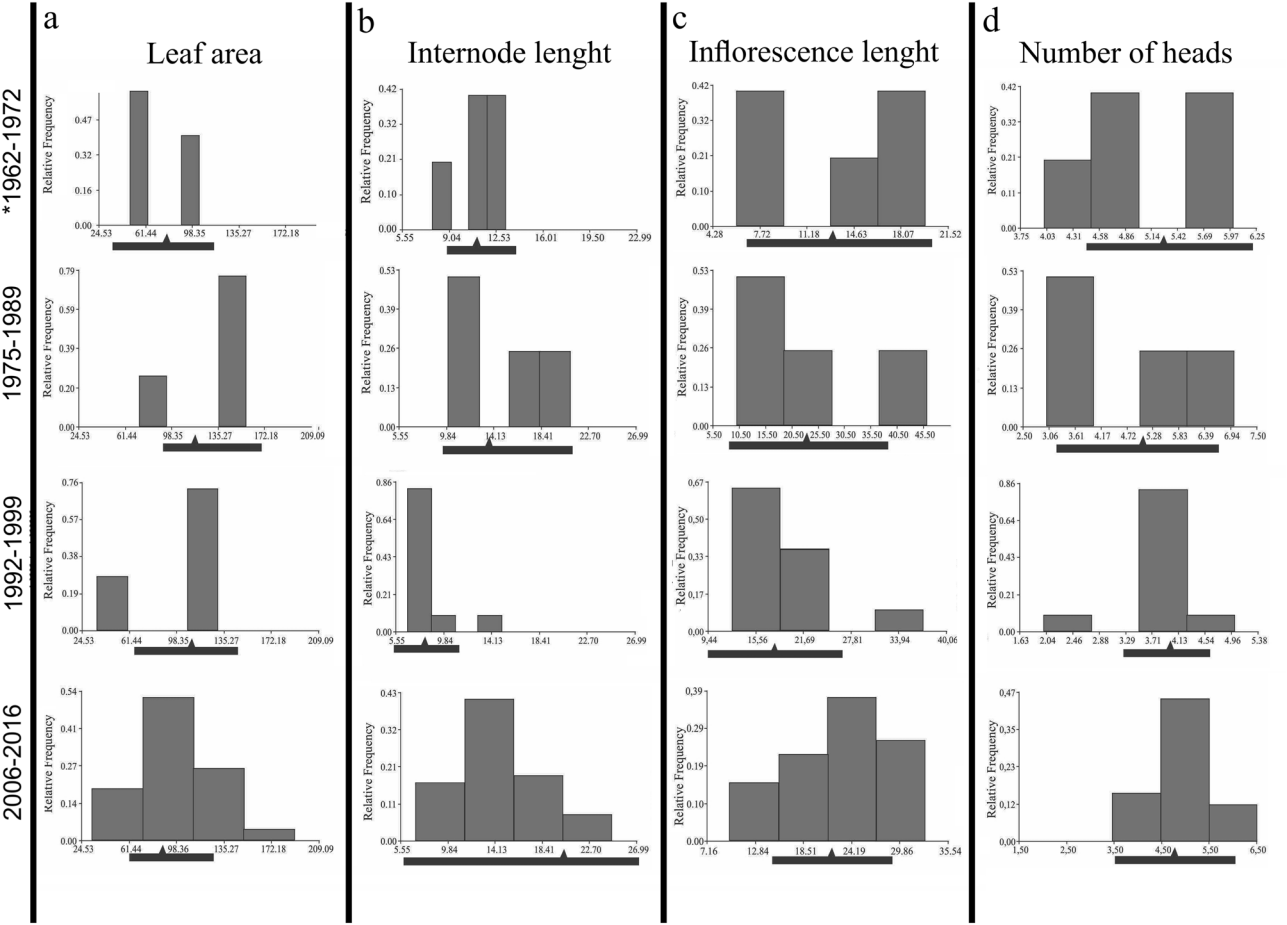
Frequency histograms of the established areas showing the action of natural selection over time.

The frequency histogram of each range shows the action of the different types of natural selection. In the established range, during the first three decades, the populations were subjected to disruptive selection, which ultimately tended to increase leaf area and length of internodes and inflorescences (Fig. 5a–c), unlike number of heads, which tended to decrease (Fig. 5d).

**Figure 6:**
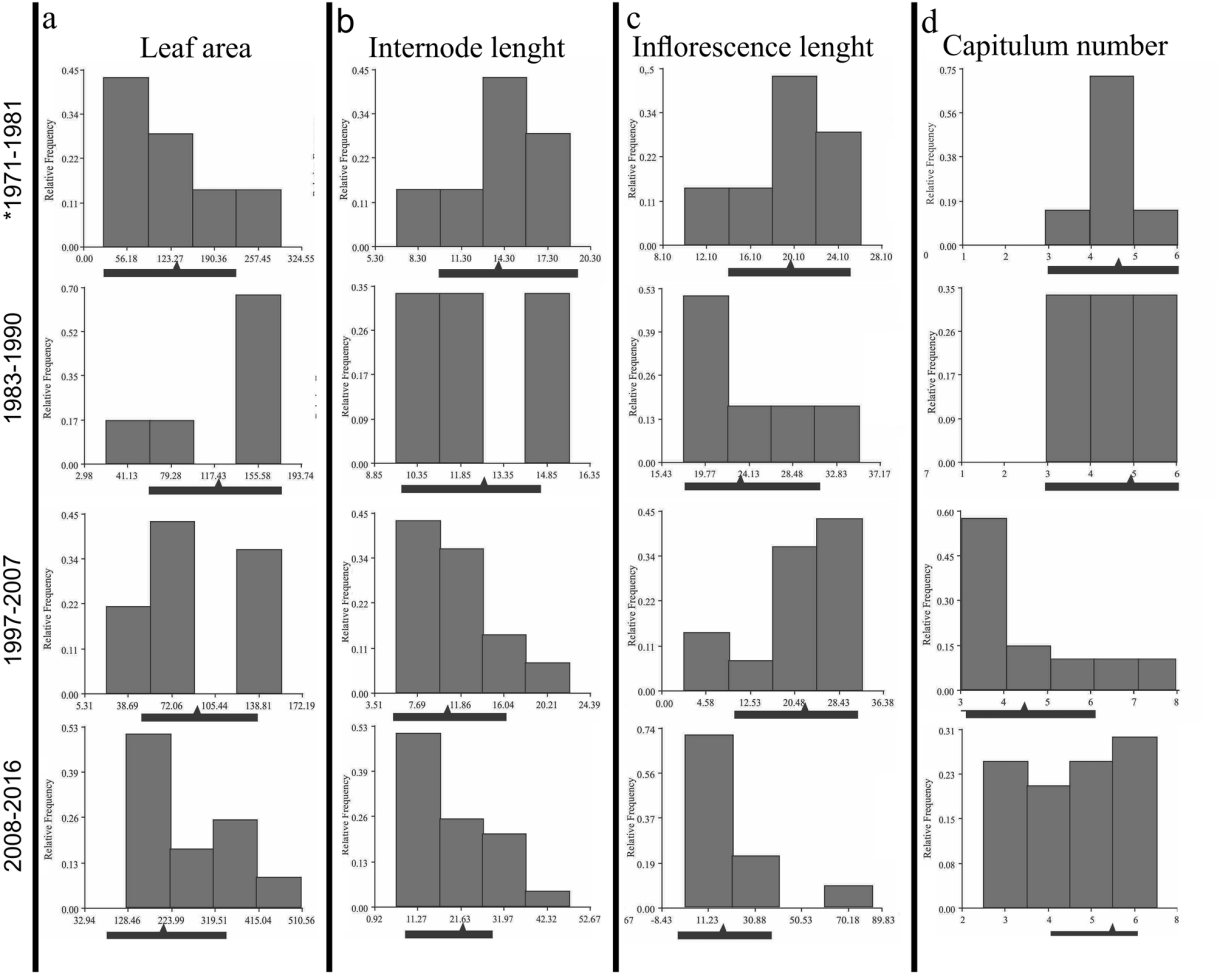
Frequency histograms of the edge areas showing the action of natural selection over time.

On the other hand, in the populations from the range edge the analyzed traits (i.e. leaf area, internode length, inflorescence length and number of heads) showed directional selection, this led to the reduction of these structures (Fig. 6a–c). However, the number of heads evidenced the opposite direction in the selection, i.e. their number increased over time (Fig. 6d).

## Discussion

The degree of morphological variation of invasive populations across a wide environmental range can be important to determine their performance and shaping forces. The morphometric analyses performed in the invasive populations of *S. madagascariensis* evidenced differences in all the measured plant traits. However, only leaf area was statistically significant among areas. This non-significant differentiation among areas can be explained mainly by gene flow, which acts by homogenizing the allelic frequencies and constraining the adaptation to a heterogeneous environment^13^.

Because phenotypic expression is the result of the interaction between genetic components and environmental pressures, neighboring areas are expected to present a greater morphological similarity due to the gene flow between them. This assumption is supported by the dendrogram of the sampled areas, since the core, source and intermediate areas are grouped in the same cluster. In addition, our results evidenced that the NWA and CBA areas had the lowest diversity of those studied which could be due to the dispersion process among the neighboring areas. This hypothesis is supported by a previous study performed in invasive populations of *S. madagascariensis*, which shows that the areas of lower altitudes (such as the source, core, intermediate and NEA) were characterized by populations with a greater dispersal ability^20^. Conversely, the areas of higher altitudes (such as NWA and CBA) presented a lower dispersal^20^.

The role of local adaptation and natural selection is often mentioned to explain the distribution range. The individuals that reached Argentina (located in the established range) were probably pre-adapted to the environmental conditions of the new range due to the rapid evolution of some traits. In this sense, the increase of leaf area could be advantageous, since water availability and temperatures in Argentina are similar to those in the native range; therefore, selection would have favored the photosynthetic capacity^25^. Regarding number of heads in the established range, the increase in the number of individuals in each population could favor their density, which could facilitate the conformation of an invasion front, particularly in *S. madagascariensis*^25^.

In the range edge, directional selection tended to reduce leaf area. These populations occur under environmental conditions characterized by drought and high temperatures; therefore, a reduced leaf area can prevent water evaporation. Likewise, length of internodes and inflorescences was also reduced, likely due to the windy conditions and higher altitudes prevailing in this region. Similar results have been reported in populations of *Senecio inaequidens* DC. along altitudinal gradients^26^, where the increasing altitude was found to negatively affect plant height and above-ground biomass. Finally, the reduction of these structures can be offset by the increase in the number of heads driven by directional selection. This compensation ensures the scattering of a greater number of diaspores.

Further, phenotypic diversity of invasive species can be affected by the number of introduction events to the new region, which increases the genetic novelties^27–29^. For example, numerous introductions of *Phalaris arundinaceae* L. to the invasive range in North America have been found to increase phenotypic and genetic diversity. Those events stimulated the rapid evolution and phenotypic plasticity, facilitating range expansion^30^. Likewise, the multiple introductions of *S. madagascariensis* in Brazil are a gene source that might ensure a higher variability in dispersal traits of the invasive populations^31^.

Nevertheless, due to the large geographical range of this species, it is likely that multiple events have occurred in Buenos Aires province. This hypothesis can explain the variability of population clusters observed in the intermediate and neighboring areas. However, molecular data are needed to determine the source of *S. madagascariensis* populations and to establish relationships with the phenotypic variation recorded across the invasive range.

In conclusion, this work constitutes a background on the invasion history of *S. madagascariensis* in Argentina. The results have shown the importance of the environment and the dispersion processes in the phenotypic variability of the sampled areas. In this context, determining this variability and the processes that affect them could be useful in order to establish priority areas of control in invasive species.

## Methods

### Plant material and sampling locations

We analyzed traits of 49 specimens collected in specific field trips and 50 specimens from vouchers of several herbaria (BA, CORD, CTES, LIL, LP, SF, SI) of *Senecio madagascariensis*; specimens span 54 years of collection efforts. The individuals are grouped in 42 populations distributed across the invasive range in Argentina (Fig. 1). In order to ensure independence among populations, we considered a group of individuals separated by at least 10 km between them as a population. The sampled areas follow the delimitations previously proposed for this invasive species in Argentina^20^. The areas located in the range edge were: NEA (Northeast of Argentina), NWA (Northwest of Argentina) and Córdoba province (CBA), whereas the remaining areas were located in the established range (intermediate, core and source).

### Morphological traits

We studied vegetative (leaf area and internode length) and reproductive (inflorescence length and number of heads) traits. Observations and measurements were performed using a stereoscopic microscope; 5–10 samples per specimen were taken for each trait.

### Statistical analysis

We performed a clustering analysis using Euclidean distances to assess the relationship among the studied populations and areas. In addition, we analyzed the morphological traits to establish the presence of significant differences through an analysis of variance (ANOVA) and a Tukey’s test (*p* < 0.05). Finally, in order to define which morphological traits are responsible for population structuring, we performed a principal component analysis (PCA). The clustering analysis and PCA were performed using the INFOSTAT statistical software^32^ and the Tukey’s test was performed using R studio software^33^.

Additionally, to determine the form of natural selection acting in the sampled areas (edge vs. non-edge), we performed frequency histograms for all the analyzed traits. Histograms were constructed considering four time windows of approximately 10 years each. Using this methodology, we considered the population introduced during the first time period as the population previous to natural selection in the new habitat, both in edge and non-edge ranges. Therefore, the deviations from the average values of the frequencies would determine the form of selection involved: stabilizing, directional or disruptive.

## Supplementary information

Supplementary Table S1.
